# Metabolome Variation between Strains of *Microcystis aeruginosa* by Untargeted Mass Spectrometry

**DOI:** 10.3390/toxins11120723

**Published:** 2019-12-11

**Authors:** Marianne Racine, Ammar Saleem, Frances R. Pick

**Affiliations:** 1 Department of Biology, University of Ottawa, Ottawa, ON K1N 6N5, Canada; ammarsal@gmail.com (A.S.); frances.pick@uottawa.ca (F.R.P.); 2 Current address: Environment and Climate Change Canada, Canada Centre for Inland Waters, Burlington, ON L7S 1A1, Canada

**Keywords:** Cyanobacteria, *Microcystis aeruginosa*, metabolome, microcystins, oligopeptides, HRMS (UPLC-QTOF)

## Abstract

Cyanobacteria are notorious for their potential to produce hepatotoxic microcystins (MCs), but other bioactive compounds synthesized in the cells could be as toxic, and thus present interest for characterization. Ultra performance liquid chromatography and high-resolution accurate mass spectrometry (UPLC-QTOF-MS/MS) combined with untargeted analysis was used to compare the metabolomes of five different strains of the common bloom-forming cyanobacterium, *Microcystis aeruginosa*. Even in microcystin-producing strains, other classes of oligopeptides including cyanopeptolins, aeruginosins, and aerucyclamides, were often the more dominant compounds. The distinct and large variation between strains of the same widespread species highlights the need to characterize the metabolome of a larger number of cyanobacteria, especially as several metabolites other than microcystins can affect ecological and human health.

## 1. Introduction

Visible accumulations of cyanobacterial biomass in the form of surface blooms have been increasing over the past few decades [1,2], mainly due to eutrophication associated with increased nutrient loading to surface waters [3,4]. In addition, other factors including global warming, changes in hydrology, and pesticide use may be acting synergistically to accelerate cyanobacterial blooms [1,5,6,7,8]. These blooms can also be harmful since cyanobacteria have the potential to produce a range of toxins, including hepatotoxins, neurotoxins, and endotoxins [9,10]. In 1996, 60 patients died due to acute neurotoxicity and subacute hepatotoxicity in Caruaru, Brazil, after hemodialysis treatments using cyanobacteria-contaminated water [11]. Recent mortality events due to the presence of one or several of these toxins have been reported in domesticated animals and diverse wildlife populations around the world, including giraffes, deer, sea otters, flamingos, and fish [12,13,14,15]. The presence of these toxins cannot be predicted from the cyanobacterial species composition and water samples need to be tested chemically in order to determine the potential toxicity and risk to human or wildlife health. Surveys worldwide suggest hepatotoxic blooms, mostly due to microcystins, are more common than neurotoxic blooms [16].

The hepatotoxic microcystins (MCs) are the most commonly studied cyanobacterial metabolites. Found in widespread genera such as *Microcystis*, *Dolichospermum* (*Anabaena*), and *Planktothrix* [17,18], MCs cause the inhibition of protein phosphatases 2A and 1 in vertebrates, which leads to an accumulation of phosphorylated proteins in cells. This accumulation can affect different metabolic pathways, including the inhibition of tumor suppressor proteins, cell proliferation and eventually, death [9,19,20,21]. Despite many ecological impacts, the biological role of MCs within cyanobacteria remains unknown, which makes their control difficult. Remarkably, the cell concentrations of MCs in toxigenic taxa can reach those of chlorophyll *a* [22], the primary pigment involved in photosynthesis in cyanobacteria, algae and higher plants.

Thus far over 269 variants have been identified with molecular weights varying between 880 and 1200 Da [10,23]. Amino acid variation in various positions of the cyclic molecule affects the degree of toxicity in vertebrates, as shown by LD_50_ values between 50 μg/kg (MC-LA) and 800 μg/kg (MC-RR) in mice after intraperitoneal injection [16]. Microcystins are thus potentially more lethal to vertebrates than several highly potent poisons, such as sarin [24]. Increasing toxic effects are observed when hydrophobic amino acids (e.g., methylated amino acids) are present in the molecule, which can facilitate MC penetration into the tissues [25]. However, the ADDA moiety of the molecule (3-amino-9-methoxy-10-phenyl-2,6,8-trimethyldeca-4,6-dienoic acid), found in all MC variants, is known to have a major role in the toxicity [25].

In addition to microcystins, cyanobacteria can produce a wide range of bioactive compounds. These include an extraordinary diversity of oligopeptides (e.g., cyanopeptolins, aeruginosins, microviridins) [26]. Several may also be bioactive or even toxic to vertebrates since many are produced through the same metabolic pathway as the MCs [27] and have similar structures [28]. Besides the public and environmental health needs for more research on these compounds, cyanobacterial oligopeptides have potential applications in the pharmaceutical, food, and energy industries [29]. Analysis of this diversity can be conducted through metabolomics, the study of the sets of metabolites produced by an organism. This approach enables detection of changes in the metabolic pathways between and within organisms in response to environmental stressors. Untargeted metabolomics (also referred to as nontarget analysis) studies the overall change of the metabolome without targeting specific compounds; it is a qualitative analysis that facilitates the detection of metabolites without prior knowledge of their presence, as well as potentially novel compounds [30]. Cyanobacteria metabolites have been studied by applying both targeted and untargeted metabolomics approaches with a major focus on microcystins [31,32,33,34,35,36,37,38,39,40] while targeted metabolomics have most commonly been applied to enable the identification of a few less studied metabolites (e.g., aeruginosins, β-methylamino-L-alanine, cyanobactins) [41,42,43,44]. This latter approach is useful but does not represent the entire diversity of metabolites at the metabolome level. Yet, Guyot et al. [26] demonstrated that over 90% of cyanobacterial metabolites were unidentified with the diversity and variation amongst species largely unknown. Few studies have applied analytical approaches that enable comparisons of ‘global’ metabolic profiles in cyanobacteria [34,45,46,47].

This study reports the application of an analytical workflow based on ultra high-performance liquid chromatography (UPLC) coupled with high-resolution accurate mass spectrometry (QTOF-MS), and pattern recognition statistical analysis to differentiate strains by their metabolome. We selected five strains from the widely distributed bloom-forming cyanobacterium, *Microcystis aeruginosa.* With the exception of PCC7806 (the wild type and its mutant *mcy*B-), the strains were isolated from eutrophic inland waters of Canada.

## 2. Results

### 2.1. Identification of Cyanobacteria Metabolites

By applying optimal chromatographic and spectrometric conditions, 2168 signals (i.e., ions detected at low fragmentation settings) were detected above the specified noise level across all strains. These signals represent the extractable and identifiable metabolomes of the organisms under study. The metabolites that could be identified are presented in Table 1. While some of them were unequivocally identified by comparison to analytical standards (e.g., Supplementary Material Figure S1), other identifications are tentative. In addition to the identified metabolites, three unidentified metabolites had major fragments in common with the MC standards while in high energy collision mode, including the fragment from the ADDA moiety at *m/z* = 135.0808 Da (Supplementary Material Figures S2–S4). They are presented as unidentified MCs, as further investigation with Nuclear Magnetic Resonance (NMR) techniques would be required to confirm their identity. Some likely candidates can, however, be identified (Supplementary Material Table S1) from the molecular weights, as previously reported by Spoof and Catherine [23]. These three unidentified metabolites were considered as microcystins for the rest of this study.

**Figure 1:**
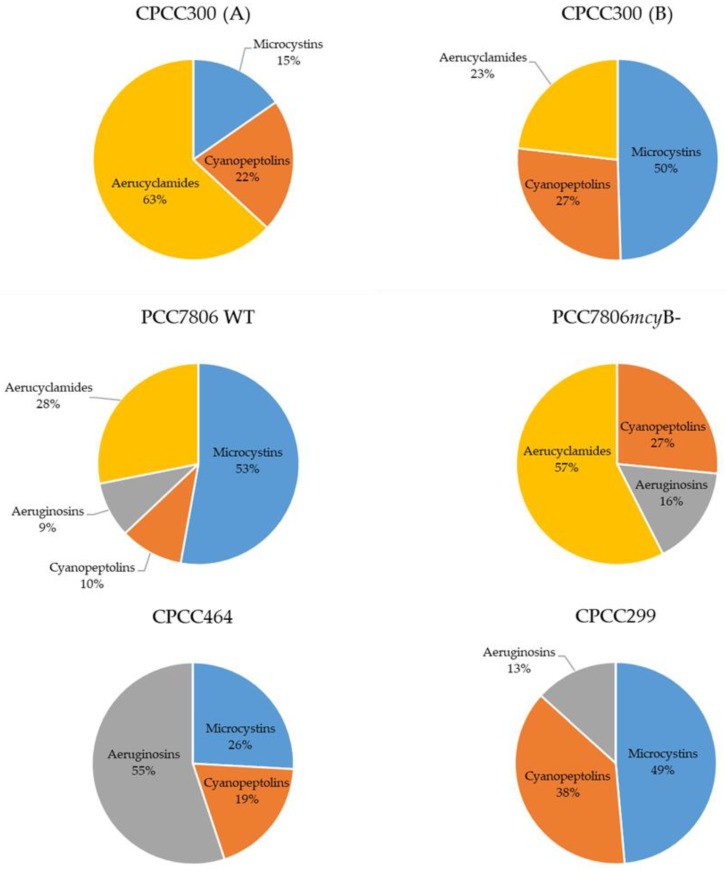
Relative amounts of oligopeptides families identified in the strains of *M. aeruginosa.* Values represent averages of biological replicates in semi-continuous cultures (CPCC464 n = 3, CPCC300 (A) n = 5, CPCC300 (B) n = 5, PCC7806 WT n = 5, PCC7806 *mcy*B- n = 4), except for CPCC299, n = 1. CPCC300 was separated into two groups to represent the two different sets of cultures that were not grown simultaneously. Unidentified metabolites were not taken into consideration.

Relative amounts of oligopeptides in the strains are presented in Figure 1. The wildtype strain PCC7806 (WT) was the only culture studied with four different oligopeptides families: microcystins, aeruginosins, cyanobactins, and cyanopeptolins, while all the others had different combinations of the three families. Samples from CPCC300 (A) and CPCC300 (B) differed in their relative metabolite composition. In CPCC300 (A), the dominant group was aerucyclamides with 63.1% (± 4.0% of one standard deviation), while microcystins were dominant in CPCC300 (B), with 49.5% (± 2.8%). While CPCC300 (A) was kept in a semi-continuous batch culture to acclimatize for eight days prior to harvesting of cells, CPCC300 (B) was kept for only five days (for reasons of logistics, and because optical densities had stabilized). For CPCC300 (B), the replication among cultures was much poorer, with one replicate similar to CPCC 300 (A) and the others deviating between the aerucyclamides and microcystins. In WT and CPCC299, microcystins represented 52.8% (± 3.0%) and 48.6% of the detected metabolites, respectively. Aeruginosins were dominant in CPCC464 (55.1% ± 3.7%) and PCC7806 *mcy*B- (57.5% ± 14.6%) (Figure 1).

**Figure 2:**
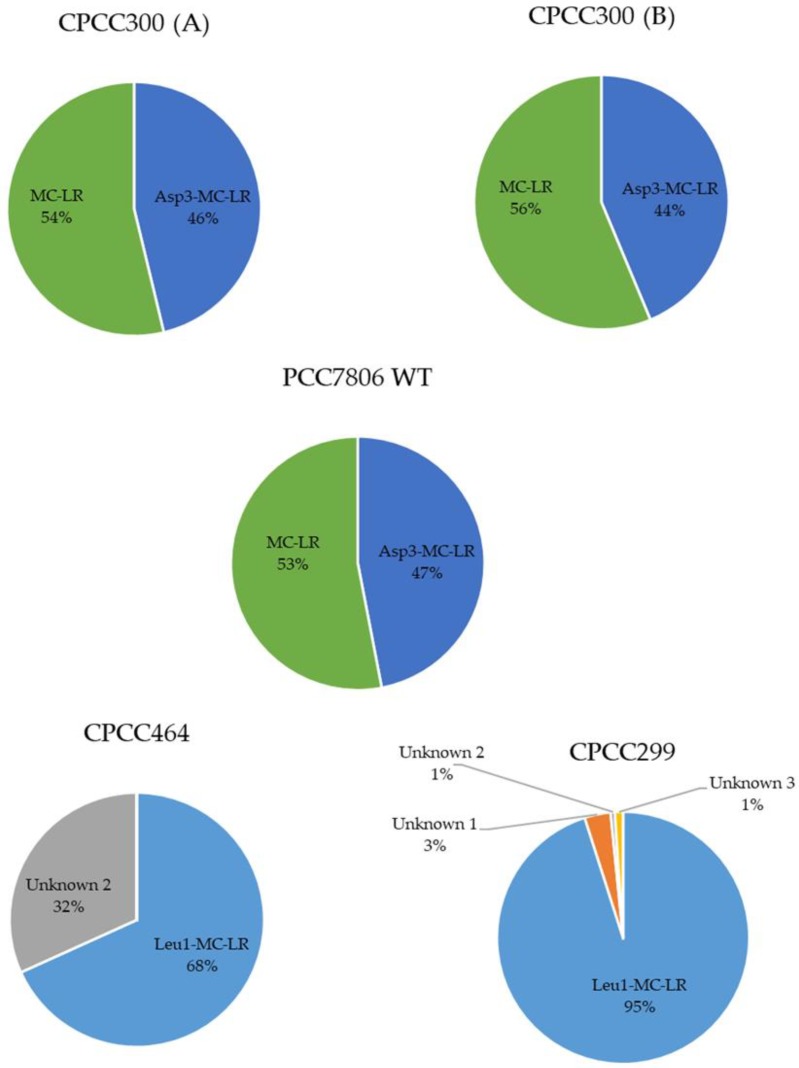
Relative microcystin variant concentrations among microcystin producing strains of *M. aeruginosa*. Values represent averages of biological replicates in semi-continuous cultures (CPCC464 n = 3, CPCC300 (A) n = 5, CPCC300 (B) n = 5, PCC7806 WT n = 5) except for CPCC299, n = 1. CPCC300 was separated into two groups to represent the two different sets of cultures that were not grown simultaneously.

**Figure A1:**
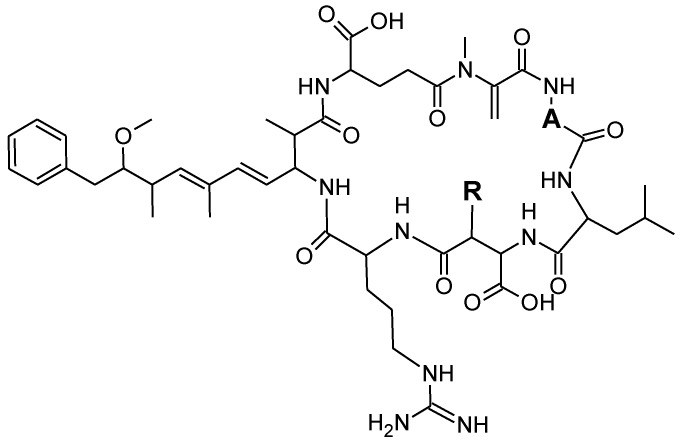
Structures of the identified microcystins variants.

**Figure A2:**
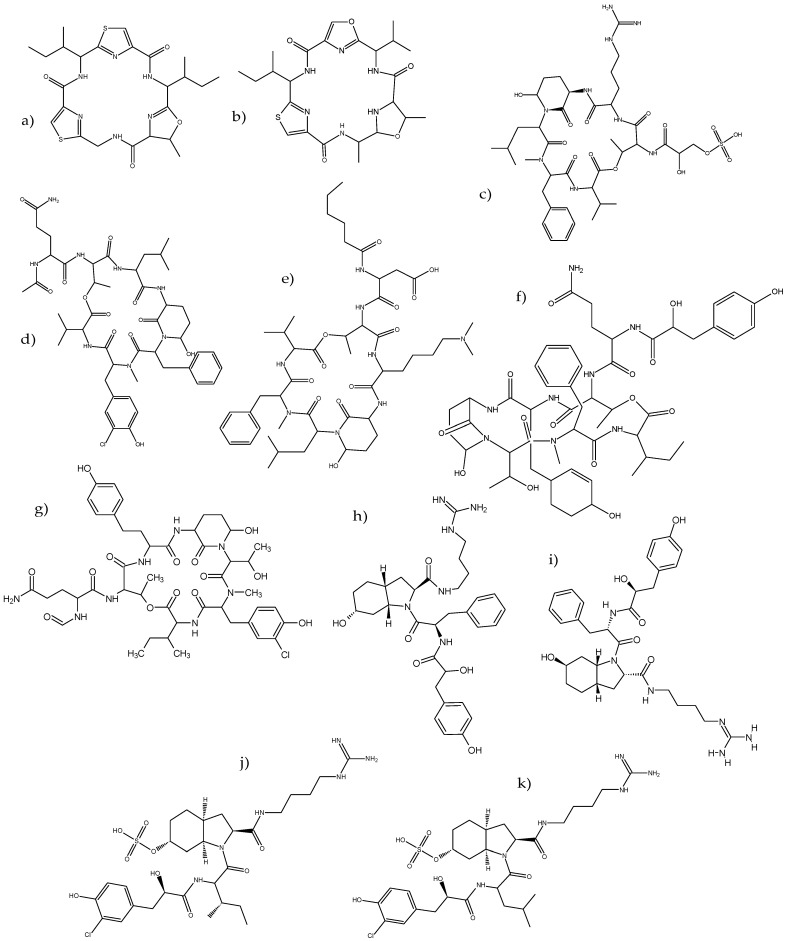
Structures of the oligopeptides (**a**) Aerucyclamide B, (**b**) Aerucyclamide C, (**c**) Cyanopeptolin 911, (**d**) Cyanopeptolin 954, (**e**) Cyanopeptolin D, (**f**) Aeruginopeptin 228B, (**g**) Anabaenopeptilide 90B, (**h**) Aeruginosin KT608, (**i**) Microcin SF608, (**j**) Aeruginosin 98A, (**k**) Aeruginosin DA688.

Amongst the different MC variants, MC-LR was the dominant variant found in CPCC300 (A) with 53.9% (± 3.9%); in CPCC300 (B) with 56.3% (± 1.7%); in PCC7806 WT with 53.0% (± 2.3%). Four different microcystins were found in CPCC299, with Leu^1^-MC-LR being the most prevalent one, at 95.1% of all MCs detected. In CPCC464, 31.8% (± 4.2%) of microcystins found corresponded to the signal of an unidentified microcystin variant (Unknown 2), while the remaining 68.2% (± 4.2%) corresponded to the Leu^1^-MC-LR variant (Figure 2). The Leu^1^-MC-LR variant was identified in both CPCC299 and CPCC464 through the detection of a signal at 1037.6033 Da, which would correspond to the protonated molecule (Figure A1) at a retention time (RT) of 3.07 min [23,48]. Under a high-energy collision mode, fragmentation was similar to that of MC-LR (Supplementary Material Figure S5), the most important fragment being from the ADDA moiety at *m/z* = 135.0808 Da. The presence of this fragment and the similar spectra provided additional evidence for the identification of this molecule as a microcystin variant. An example of fragmentation of MC-LR and the corresponding signals in the positive ionization mode is presented in Supplementary Material Table S2. Another molecule identified with the fragmentation pattern was a type of cyanopeptolin in *M. aeruginosa* CPCC299 and CPCC464. For this compound, two other major peaks were observed at RT 2.13 and 2.25 min (Supplementary Material Figure S6), both presenting the same signals corresponding to the cyanopeptolin variant CPT911 (Figure A2). Masses corresponding to fragments of the molecule were found in both signals (Supplementary Material Table S3). The two peaks could be explained by the presence of an isomer in the analyzed mixture. The higher signal intensity of the first peak (CPT911A) might reflect the dominance of this isomer. Both signals were, however, well above the S/N threshold ratio.

### 2.2. Metabolomes of M. aeruginosa Strains

**Figure 3:**
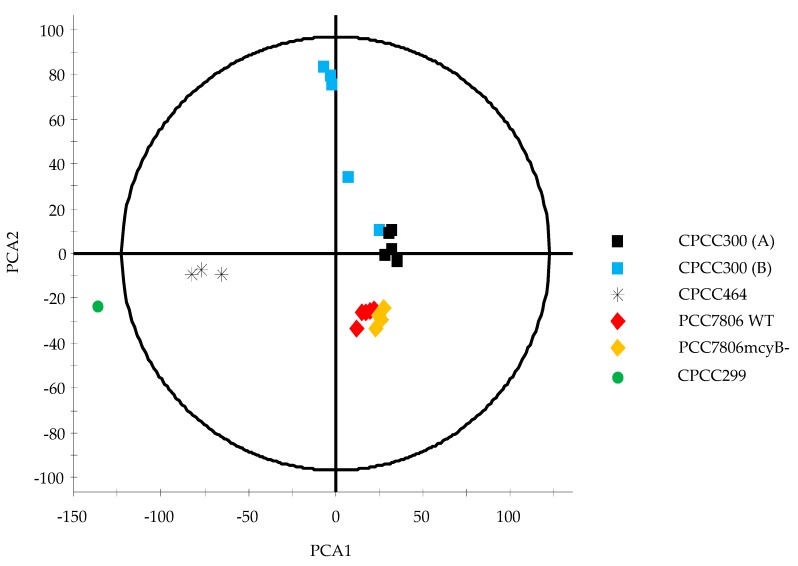
Principal component analysis (PCA) scores plot of *M. aeruginosa* strains. Each point represents all extractable and detectable metabolites in the samples (CPCC300 (A) n = 5, CPCC300 (B) n = 5, CPCC464 n = 3, CPCC299 n = 1, PCC7806 WT n = 5, PCC7806 *mcy*B- n = 4). PC 1, on x-axis, explains 27.84% of the variation between strains. PC 2 explains 17.32% of the variation. Hotelling’s ellipse for 95% confidence excludes the CPCC299 profile.

**Figure 4:**
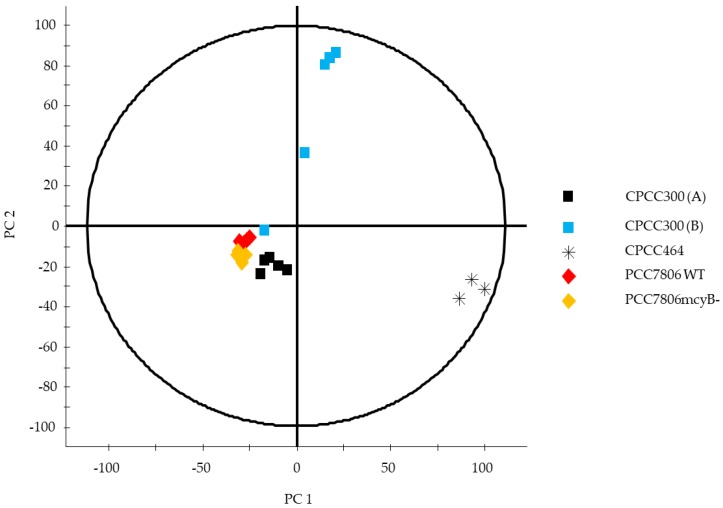
PCA scores plot of *M. aeruginosa* cultures, excluding CPCC299. Each point represents the combination of metabolites found in one sample. PC1 explains 25.27% of the variation between strains. PC2 explains 20.08% of the variation. Hotelling’s ellipse represents the 95% confidence interval.

An unsupervised multivariate analysis was conducted by Principal Component Analysis (PCA) (Figure 3), a mathematical procedure used to reduce large sets of variables to smaller ones by creating new variables named “Principal Components” (PCs), a PCA summarizes the total variance in the data and is used to examine tendencies rather than being a formal statistical analysis. In the absence of replicates for CPCC299 and to ensure no bias was introduced in the analysis by its presence, a Hotelling’s ellipse for 95% confidence was applied [49]. It identified CPCC299 as an outlier by its presence outside the 95% confidence range. A second PCA was conducted without this strain. By excluding CPCC299, the metabolic separation between the other strains was more evident (Figure 4). 

The strains were separated from each other along the first axis (PC1), which explained 25.27% of the overall variation and represented the change in metabolite composition and concentration between the strains. The lower variation with respect to PC2 (20.08%) indicated that the relative metabolite composition was more similar between strains than within the strains. The largest variation observed along PC2 was found within the CPCC300 (B) samples, but the variation among replicates was very high, with one replicate close to those of the cultures acclimated for longer under semi-continuous growth conditions (CPCC300 A). 

**Figure 5:**
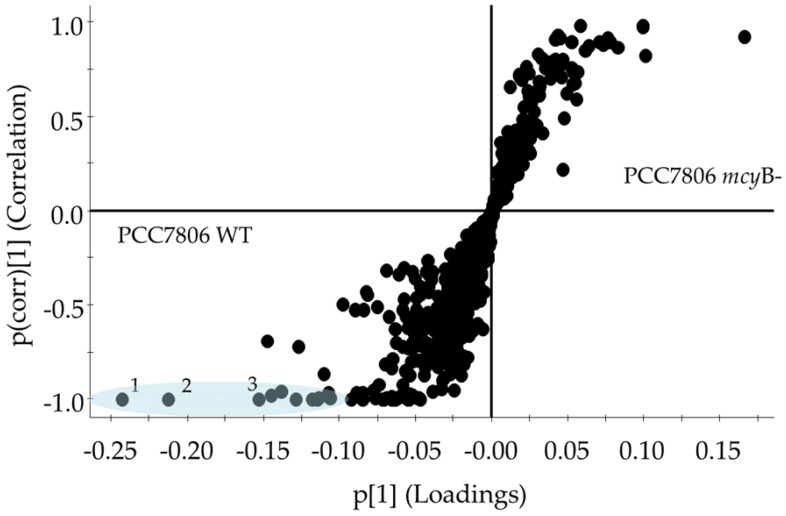
Supervised scattered loadings plot (i.e., S-plot) for metabolome comparison between the mutant *mcy*B- and the wildtype (WT) cultures of *M. aeruginosa* PCC7806. PCC7806 *mcy*B- metabolites are on the right (y axis 0 to 1) while the WT are on the left (y axis −1 to 0). Each dot represents an exact mass and RT pair. The further at the extremity of the “S” shape, the greater the confidence of variation between the two cultures. Metabolites for which a high correlation and high confidence were found are highlighted in blue, and three numbered fragments correspond to MC-LR (1) 502.2640, 2.84 min; (2) 509.2705, 2.84 min; (3) 135.0802, 2.85 min. Molecular formulas for the fragmentation are available in Table S2.

**Figure 6:**
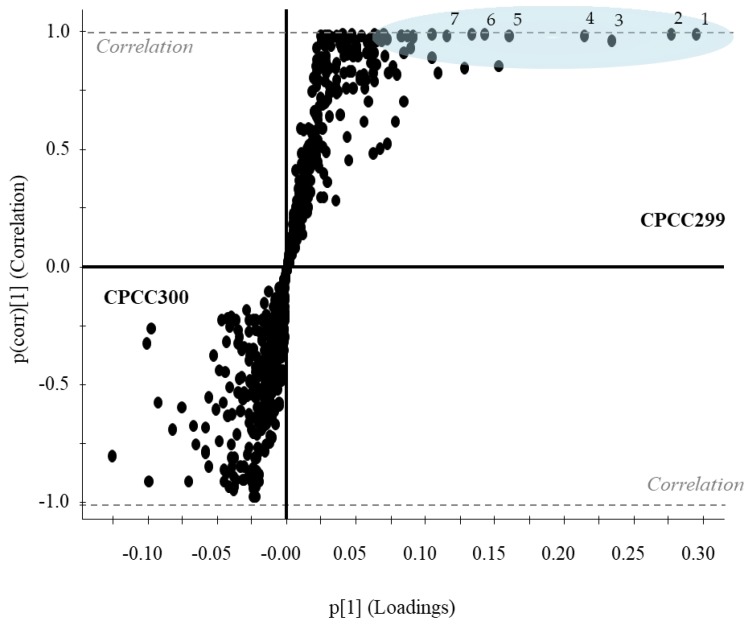
S-plot for metabolome comparison between strains CPCC300 and CPCC299 of *M. aeruginosa*. CPCC299 metabolites are on the right (y axis 0 to 1) while the CPCC300 are on the left (y axis −1 to 0). Each dot represents an exact mass and RT pair. The further at the extremity of the “S” shape, the greater the confidence of variation between the two cultures. Metabolites for which a high correlation and high confidence were found are highlighted in blue: (1) 365.0870, 045 min; (2) 515.3174, 5.54 min; (3) 277.0853, 0.45 min; (4) 220.0825, 1.04 min; (5) 409.1606, 3.88 min; (6) 677.3703, 5.03 min; (7) 530.3157, 5.31 min.

In order to examine which metabolites contributed to the differences in the metabolomes, orthogonal partial least-squares discriminant analysis (OPLS-DA) was performed. OPLS-DA classifies and separates the data into two classes and investigates the factors responsible for this separation [49]. It is visualized by an S-plot, which is a loadings plot that provides a graphical summary of the correlation between X and Y [50]. Each dot represents a unique RT and accurate mass pair. The placement of a dot in S-plot further from the core on the x-axis with the highest correlation to the strain (y-axis) can be considered discriminant. This statistical approach is suitable for handling raw LCMS data for untargeted metabolomics workflows [51]. The S-plot between the strains WT and *mcy*B- (mutant) showed three fragments at a RT of 2.84 min (Figure 5) in WT only. These signals correspond to MC-LR, as confirmed by a standard, and were completely absent from the *mcy*B- strain, as expected. Another S-plot comparing CPCC299 and CPCC300 (Figure 6) indicated that a significant number of fragments distinguished these two strains and were responsible for the strong differentiation observed in Figure 3.

## 3. Discussion

### 3.1. Metabolome Diversity in M. aeruginosa

The selected strains of *M. aeruginosa* varied widely in their metabolome. When using targeted and non-targeted (PCA, OPLS-DA) analyses, CPCC464 and CPCC299 strains appeared to differ the most in their metabolomes (Figure 1, Figure 2 and Figure 3). Further investigation would be required beyond mass spectrometry in order to identify the nature of the discriminant fragments of CPCC299 (Figure 6). It is interesting to note that all peptides found in the strains CPCC464 and CPCC299 are metabolites known to be produced non-ribosomally (microcystins, cyanopeptolins, aeruginosins) [28,41] while CPCC300 and PCC7806 (WT and mutant) had peptides from both the ribosomal (aerucyclamides) and non-ribosomal pathways [52]. The differences observed among the strains could be explained by this variation in the expressed pathways. There was also some variation in the relative metabolite composition observed between the two groups of CPCC300, with a larger amount of microcystins produced in the B cultures grown later, despite similar environmental conditions. There was evidently more variability and some bias introduced by the shorter acclimation period used to stabilize growth rates. This result illustrates the need to ensure constant growth rates in similarly acclimatized replicated cultures in order to make inferences about differences in metabolomes and, by extension, to compare results across studies of different cyanobacteria. However, environmental growth conditions such as temperature and light exposure were less important than underlying genetic differences between strains in determining overall metabolite profiles. 

In the wild-type PCC7806, more than half of the oligopeptides detected (52.8% ± 3.0% of one standard deviation) were microcystins (two variants, MC-LR and [Asp^3^]-MC-LR, Figure 1 and Figure 2). If we exclude this class from the analysis, the amounts of the metabolites remaining corresponded proportionally to that of the mutant strain, PCC7806 *mcy*B-. The *mcy*B mutation therefore does not affect other peptides, which supports earlier studies of these strains based on targeted analyses of selected peptides [53].

### 3.2. Identification of Metabolites

The large number of signals detected in each culture corresponded to as many metabolite fragments at various RTs. Most of these could not be identified (500-900 per sample), and the few identifications without validation via standards were tentative. Analytical standards in mass spectrometry techniques are required in order to compare with the retention and fragmentation data, but standards are only available for the most abundant microcystins and a few other metabolites. For example, no aeruginosin is currently available as a commercial standard. Metabolites would need to be extracted and sufficiently concentrated from pure culture to be analyzed by NMR [42]. Many unidentified oligopeptides were found in the MC-producing strains CPCC299, CPCC300, PCC7806 and in the mutant PCC7806 *mcy*B-. However, fewer were observed in the CPCC464 strain (Table 1). Nevertheless, CPCC464 appeared to produce several different and undescribed microcystins, as in a previous analysis [54], and the absence of other peptides in the sample seems unusual.

Aside from MC-LR and closely related variants [Asp^3^]-MC-LR and [Leu^1^]-MC-LR, three unconfirmed variants of microcystins were detected. Those metabolites were identified as microcystins because of the presence of the ADDA fragment (Supplementary Material Table S2) under the high energy collision mode ([M + H] = 135.0808 Da) which characterizes microcystins. A few other signals were observed in the spectra, which were similar to those of the MC standards available and could be a product of microcystin fragmentation. Further investigation of those spectra could confirm identifications, although several possibilities were pinpointed by comparing the pseudo-molecule masses in the spectra to the theoretical values of known MC variants [23].

Despite their presence in the toxigenic strains, microcystins were not always the dominant oligopeptides produced by *M. aeruginosa* (Figure 1). A few specific metabolites known to be produced by cyanobacteria, and more particularly *Microcystis,* were detected. A vast majority can be described as oligopeptides, but the amino acid intermediate N-acetylornithine was also detected in CPCC300 and CPCC464. N-acetylornithine is involved in the arginine biosynthetic pathway, which plays a part in nitrogen fixation in cyanobacteria [55] and the biosynthesis of secondary metabolites. 

Cyanobactins, cyanopeptolins, and aeruginosins were the oligopeptide families with an equal or greater importance in the various strains (Figure 1). With more than a hundred variants identified to date [52], cyanobactins are considered the second largest class of metabolites in cyanobacteria in terms of different variants, after microcystins. They are also cyclic peptides, but are characterized by the presence of heterocyclized amino acids, or isoprenoid derivatives. The cyanobactins aerucyclamides identified in this paper have been previously identified in some *M. aeruginosa* strains [52]. They have been shown to bioaccumulate, to be toxic to freshwater crustaceans, as well as having potential antiparasite and antimalarial properties [52,56]. 

Cyanopeptolins (CPT) include more than 82 variants [28], as variation occurs at almost every amino acid present, except for the Ahp residue that characterizes the peptide (3-amino-6-hydroxy-2-piperidone) and the threonine [28]. However, little is known of their production, regulation and uptake by vertebrates [57]. They were initially considered non-toxic [31], but it has recently been shown that some variants have toxic effects on zebrafish [57] and zooplankton [58]. 

Aeruginosins (ARG) are protease inhibitors produced non-ribosomally in *M. aeruginosa*, *M. viridis* and *Planktothrix* (*Oscillatoria*) *agardhii* [41] and characterized by the presence of two uncommon residues, Hpla ((4-hydroxy)phenyllactic acid) and Choi (2-carboxy-6-hydroxyoctahydroindole). They include microcins and spumigins, which brings the number of identified variants to 27 [28], but there is potential for a greater number as considerable variation is observed among all amino acids in the molecule [42].

Some other metabolites were expected in the *M. aeruginosa* strains but were not found or were below the detection limit. For example, β-cyclocitral, a compound produced in several *Microcystis* species [59], was not detected in any sample. This could be due to its volatile nature that would be easier to detect with a GC-TOF or GC-triple quadrupole [59]. Moreover, the natural amino acid β-methylamino-L-alanine (BMAA), a neurotoxin, was not detected in any of the strains here, while its occurrence in freshwater cyanobacteria appears to be widespread [43]. BMAA was previously discovered in the PCC7806 strain [60] also tested in this study. The explanation probably lies in the concentration or extraction process. BMAA recovery is optimal with the use of nitrocellulose or nylon filters rather than glass fiber filters used here, which tend to adsorb BMAA and other polar molecules [43,61]; BMAA extraction is also usually conducted in water [62], because of the polarity of the molecule. In this study, a 75% methanol solution in water was used to optimize the extraction of microcystins. Also among expected molecules, Briand et al. [45] detected a new variant of cyanopeptolin, CPT895, with a pseudo-molecule [M + H] of 896 Da in PCC7806. However, no trace of this compound was found in the present study using the same strain. Briand et al. [45] used dichloromethane and methanol, which is a less polar mixture. Other studies have used various concentrations of methanol for extractions [57,58,63], and this inevitably would affect metabolome profiles. These differences in extraction protocols make direct comparisons between studies of the cyanobacterial metabolome problematic. 

## 4. Conclusions

Untargeted mass spectrometry is a new approach to identify and characterize the metabolome profiles of various species and strains of cyanobacteria. In this research, we showed how the metabolome differs significantly between strains of *Microcystis aeruginosa*, and to a lesser extent within the same strain with time factors. The latter result points to the need to ensure sufficient and similar acclimation periods for cell growth under identical environmental conditions. Unsupervised multivariate analysis (i.e., OPLS-DA and S-plot) is useful for metabolome comparison and can point to novel biomarkers to differentiate strains or changes in metabolic pathways. Untargeted mass spectrometry should be used in the future, along with NMR techniques for the discovery and characterization of new molecules. 

## 5. Material and Methods

### 5.1. Culturing Techniques

CPCC300 (300), CPCC464 (464) and CPCC299 (299) were *M. aeruginosa* strains purchased from the Canadian Phycological Culture Collection (CPCC—University of Waterloo, Waterloo, Ontario, Canada), while *M. aeruginosa* PCC7806 (WT) and PCC7806 *mcy*B- (*mcy*B-) were purchased from the Pasteur Culture Collection (PCC—Institut Pasteur, Paris, France). The strains were chosen based on their capacity to produce microcystins, and the possibility of a mutation in the case of the PCC strain. All cultures were grown in BG11 medium [64] diluted from a stock solution provided by the CPCC. A supplement of 10 μm of NaHCO3 was added to the media for PCC cultures to optimize growth conditions as suggested by the PCC. All cultures were kept in a growth chamber (Conviron E15, Winnipeg, Manitoba, Canada) under white light (50 μm m^−2^·s^−1^), with a light and dark cycle of 12 h at 20 °C. Prior to experiments, three to five replicates of each strain were grown and maintained separately in semi-continuous culture to maintain a constant growth rate (hence the physiological state in exponential growth) as monitored daily by optical density at 750 nm (Cary 100 Bio Spectrophotometer, Varian, Winnipeg, Manitoba, Canada). All replicates for strains 300 (A), WT and *mcy*B- were kept in a semi-continuous state for eight days, while all replicates for 300 (B) and 464 were kept in this state on a separate occasion for five days prior to harvesting cells for analyses (for logistical reasons). This was sufficient time to allow for stabilization of optical density. Strain characteristics are presented in Table 2.

### 5.2. Extraction

Each biological replicate was sub-sampled once (20 mL) and filtered on pre-dried (60 °C for 4 h) and pre-weighed (Mettler Toledo Balance, Mississauga, Ontario, Canada, ± 1 mg) glass fiber filters (GF/C, Whatman, Mississauga, Ontario, Canada, ~1.2 μm) and frozen at -20 °C until further analysis. Samples were dried at 60 °C for 4 h and the mass taken. Re-hydration and freeze thaw cycles were used to increase cell lysis, as the original extraction protocol suggested [65]. The particulate samples were used to extract metabolites with accelerated solvent extraction, ASE (Dionex, ThermoScientific, Oakville, Ontario, Canada), following the method described for microcystins by Aranda-Rodriguez et al. [65]. The filters were placed in 11 mL stainless steel cells lined with cellulose filters and packed with HydroMatrix (Varian Inc., Walnut Creek, CA, USA) before being extracted twice by a 75% methanol solution (FisherBrand, Ottawa, Ontario) under high pressure (2000 psi) and high temperature (80 °C) for 5 min. The 20 mL-extract was evaporated to dryness under nitrogen pressure and heated at 60 °C using a TurboVap LV (Caliper, Hopkinton, MA, USA) and re-suspended in 2 mL of 100% methanol (LC-MS grade). The solution was evaporated to dryness a second time under nitrogen pressure, and re-suspended in 50% methanol (LC-MS grade) for a final volume of 1 mL. The solution was filtered on polycarbonate filters (0.2 μm) and kept in a 1.5 mL amber glass vial at -80 °C until ready to process. For CPCC299, some samples were lost in extraction steps. 

Commercially available microcystins (9 in total) and nodularin were included in this study. Nodularin standard (CAS 118399-22-7) and MCs standards for LR (CAS 101043-37-2) and LA (CAS 96180-79-9) variants were bought from Cayman Chemicals (Burlington, Ontario, Canada). MC-LF (CAS 154037-70-4), MC-LW (CAS 157622-02-1), MC-LY (CAS 123304-10-9), MC-RR (CAS 111755-37-4), MC-WR (CAS 138234-58-9), MC-YR (CAS 101064-48-6) and [Asp^3^]-LR variants were bought from Enzo Life Sciences (Farmingdale, NY, USA). In addition, an MC standard for the variant [Dha^7^]-LR (CRM-dmMCLR, Lot 20061116) was provided by Dr. S. Sauvé, Département de Chimie, Université de Montréal. All 12 standards had a purity over 95%, and in each case 10 ppm in methanol was prepared as identification references. The oligopeptide nodularin has been used in previous studies as an internal standard to estimate the recovery of microcystins during extraction [32,65,66]. It has not been found to date in freshwater cyanobacteria or freshwater environmental samples and appears restricted to the marine environment. 

### 5.3. LC-MS Analyses

All LCMS analyses were performed on a UPLC-QTOF system (Waters Inc., Milford, MA, USA). LCMS system was comprised of an ultra-high-performance liquid chromatograph, upper pressure limit 1200 bars (Acquity UPLC), connected to a quadrupole time of flight mass spectrometer (Xevo G2 QTOF). Chromatographic separations were performed on an Acquity UPLC BEH C18 column (2.1 × 50 mm, i.d. 1.7 μm, Waters Corp. (Milford, MA, USA). LCMS grade mobile phases were (A) acetonitrile and 0.1% formic acid, and (B) water with 0.1% formic acid. Mobile phases were delivered at 0.8 mL/min with a linear gradient of 5% to 95% A in five minutes at column oven temperature of 50 °C. 

QTOF was operated at unit resolution. Two separate injections (0.2 μL) were performed to obtain positive and negative ions at collision energies of 6 V and 20–50 V to obtain low and high fragmentation spectra of each signal that was above the specified noise level. Other QTOF parameters were: scan time 0.1 s; sample cone voltage 40 V; capillary voltage 1.2 kV for positive ionization, and 2.5 kV for negative ionization; extraction cone 4-35 V; source temperature, 150 °C; desolvation temperature, 500 °C; desolvation gas flow, 1200 L/h; cone gas flow, 20 L/h. MS data were collected in scan mode from *m/z* 50 to *m/z* 1500 Da. All the data were acquired without mass correction using MassLynx V4.1 SCN918.

### 5.4. Pattern Recognition Statistical Analyses

Data were collected on the QTOF between 0.3–6 min RT and exported to MarkerLynx to generate a matrix consisting of RT, accurate mass pairs, and peak heights. These raw data were then transferred to EZInfo^TM^ (V 8.03) to generate unsupervised (i.e., PCA) and supervised (i.e., OPLS-DA) plots [49]. For principal component (PCA) and orthogonal partial least-square discriminant (OPLS-DA) analyses, the following optimized parameters were applied: mass range 50—1500; extracted ion window—0.05; apply smoothing—no; signal intensity threshold—1000; mass window—0.05; RT window—0.2 min; noise elimination, level—8. Elemental composition criteria were mass tolerance—5.0 ppm; electron state—even electrons; double bond equivalence range—-0.5 to 50.0; element number ranges—C = 40–60, H = 50–150, N = 0–20, O = 0–50, S = 0–6. The mass exclusion list included 54 ions that were observed in blank injections and were considered background signals.

### 5.5. Metabolite Identification

The identification of microcystin variants in cell culture extracts was performed by matching the (1) RTs, (2) elemental compositions, (3) parent ions, and (4) product ions. METLIN (Scripps Center for Metabolomics: https://metlin.scripps.edu) was used. For tentative identification of metabolites mass accuracy threshold was set at ± 5 ppm for the parent ion and ± 10 ppm for the respective product ions and finalized by mass fragment and ChemDraw (PerkinsElmer Informatics, Waltham, Massachusetts, USA) or as seen in available standards of similar metabolites. To search for unidentified microcystins in the samples, the total ion chromatograms (TIC) were scanned for the ADDA moiety molecular weight (structure available in Table A1), as it is specific to this group of metabolites. The mass spectra were analyzed to find other possible fragments, and a plausible pseudo molecular ion [M + H].

## Figures and Tables

**Table 1:** Metabolites identified in *M. aeruginosa* strains. Identifications confirmed by the use of a standard are noted by a (*). Structures are presented in Figure A1 and Figure A2. Possible elemental compositions are available in Table S1 for the metabolites indicated by (**).

Oligopeptide Families	Metabolites	Molecular Formula	Precursor Ion	Major Fragments	RT (min)	CPCC464	CPCC299	CPCC300	PCC7806	PCC7806 *mcy*B-
**Microcystins**	[Asp^3^]-MC-LR *	C_48_H_72_N_10_O_12_	981.5220	135.0820, 502.2665, 847.4663	2.84			X	X	
	[Leu^1^]-MC-LR	C_52_H_80_N_10_O_12_	1037.6033	135.0808, 169.1345, 530.2979, 903.5250	3.06	X	X			
	MC-LR *	C_49_H_74_N_10_O_12_	995.5287	135.0784, 213.0799, 509.2578, 861.4597	2.84			X	X	
	Microcystin (Unknown 1)	N/A**	1031.4791	135.0808, 282.1383	3.18		X			
	Microcystin (Unknown 2)		1055.5521	135.0808, 539.2790	2.98	X	X			
	Microcystin (Unknown 3)		1009.4980	135.0975, 282.1452, 404.1939	3.24		X			
**Cyanobactins**	Aerucyclamide B	C_24_H_32_N_6_O_4_S_2_	533.1835	140.0681, 337.2317, 437.1928	4.59			X	X	X
	Aerucyclamide C	C_24_H_32_N_6_O_5_S	517.2213	140.0657, 425.2299, 437.1971	4.47			X		
**Cyanopeptolins**	Cyanopeptolin 911 (CPT911)	C_39_H_61_N_9_O_14_	912.4331	157.0907, 199.1181	2.13	X	X			
	Cyanopeptolin 911 (isomer)				2.25	X	X			
	Cyanopeptolin 954 (CPT954)	C_46_H_63_ClN_8_O_12_	955.4891	222.1122, 486.2253, 915.4907	3.62		X			
	Cyanopeptolin D (CPTD)	C_48_H_76_N_8_O_12_	957.5405	222.1091, 261.1078, 470.2648	3.13			X	X	X
	Aeruginopeptin 228B	C_52_H_72_N_8_O_15_	1049.4680	129.0720, 222.1122, 404.1939, 522.2386, 888.4375	2.79		X			
	Anabaenopeptilide 90B	C_45_H_61_ClN_8_O_14_	973.4313	140.0657, 181.1215, 222.1122, 973.4313	3.47	X	X			
**Aeruginosins**	Aeruginosin KT608A/KT608B/Microcin SF608	C_32_H_44_N_6_O_6_	609.2664	149.0242, 178.1235, 242.2856, 301.1415, 515.3187	5.53	X				
	Aeruginosin KT608A/KT608B/Microcin SF608		609.3372	113.9631, 178.1225, 222.1122, 260.0569	1.65	X				
	Aeruginosin 98A/DA688	C_29_H_45_ClN_6_O_9_S	689.2940	609.3372, 280.2124	1.46	X	X			
	Aeruginosin 98A/DA688		689.2833	113.9631, 178.1225 222.1152, 481.2542	1.28				X	X
**Other**	N-acetylornithine	C_7_H_14_N_2_O_3_	175.1187	152.9637	0.40	X		X		

**Table 2:** *M. aeruginosa* strains characteristics. Maximum growth rates under the growth conditions described in Section 5.1 were calculated from optical density changes.

Strain (Abbreviation)	Origin	Max Growth Rates (d^−1^)	MC Producer
**CPCC300**	Deposited by E. Prepas/A. Lam. Isolated by A. Lam from Pretzlaff Pond, Alberta, Canada, Aug. 1990.	0.600	Yes
**CPCC464**	Deposited by D. Parker. Isolated by E.A.D. Allen/P. Gorham from Trampling Lake, SK, Canada, Aug. 1980.	0.048	Yes
**CPCC299**	Deposited by E. Prepas/A.Lam. Isolated by A. Lam from Pretzlaff Pond, Alberta, Canada, Aug. 1990	0.024	Yes
**PCC7806 (WT)**	Deposited by F.I. Kappers. Isolated from Braakman Reservoir, Netherlands, 1984.	0.024	Yes
**PCC7806 *mcy*B- (*mcy*B-)**	Deposited by E. Dittman, 1997. Genetically modified from PCC7806.	0.072	No

## Supplementary Materials

The following are available online at https://www.mdpi.com/2072-6651/11/12/723/s1, Figure S1. Separation of standards in a positive ionization mode a) MC-LR (2.87 min); b) [Asp^3^]-MC-LR (2.88 min). Figure S2. Unknown one separation chromatogram at RT 3.19 min (a), and spectra under high energy (b) and low energy (c) collision in positive ionization mode. Figure S3. Unknown two separation chromatogram at RT 2.98 min (a), and spectra under high energy (b) and low energy (c) collision in positive ionization mode. Figure S4. Unknown three separation chromatogram at RT 2.97 min (a), and spectra under high energy (b) and low energy (c) collision in positive ionization mode. Table S1. Possible MC variants for the three unknown metabolites from M. aeruginosa presented in Table 1. Figure S5. (a) Chromatography of [Leu^1^]-MC-LR variant; b) high energy spectrum of [Leu^1^]-MC-LR; c) high energy spectrum of the standard solution of MC-LR. Figure S6. CPT911 detection in *Microcystis aeruginosa* CPCC464; a) Separation of two isomers, CPT911A at RT 2.13 min and CPT911B at RT 2.25 min; b) High energy spectra for CPT911B. Table S2. Major fragments of MC-LR detected in the standard solution at a RT of 2.84 min. Table S3. Major fragments of CPT911 detected in the samples at RT 2.13 min and 2.25 min.

## Appendix A

**Table A1:** Structural variations between microcystin variants.

Metabolites	A	R
**MC-LR**	D-Ala	CH_3_
**[Leu^1^]-MC-LR**	D-Leu	CH_3_
**[Asp^3^]-MC-LR**	D-Ala	H
