# Supplementary material for: Metabolome Variation between Strains of Microcystis aeruginosa by Untargeted Mass Spectrometry
Source: Toxins (Basel). 2019 Dec 11;11(12):723. doi: 10.3390/toxins11120723 (PMC6950387; doi:10.3390/toxins11120723)
Supplement: Supplementary file 1 [file toxins-11-00723-s001.pdf]

Metabolome Variation between Strains of *Microcystis aeruginosa* by Untargeted Mass Spectrometry

Marianne Racine, Ammar Saleem and Frances R. Pick

Table S1. Possible MC variants for the three unknown metabolites from *M. aeruginosa* presented in Table 1. Corresponding molecular weights are expressed in Daltons (Da). List of MC variants from Spoof & Catherine (2017).

MC Variants	[M+H]	Molecular Formula
**Unknown 1**		
[Asp^3]MC-M(O_2)R *(oxidation artefact)*	1031.4865	$C_{47}H_{71}N_{10}O_{14}S$
[d-Asp^3, Dha^7]MC-HtyR<br>[Asp^3, DMAdda^5]MC-HtyR<br>[Asp^3]MC-RY<br>[Dha^7]MC-RY<br>[Asp^3, Dhb^7]MC-RY<br>[d-Asp^3]MC-YR<br>[Dha^7]MC-YR<br>[Asp^3, (E)-Dhb^7]MC-YR<br>[DMAdda^5]MC-YR	1031.5197	$C_{51}H_{71}N_{10}O_{13}$
**Unknown 2**		
[Met^1]MC-LR	1055.5594	$C_{51}H_{79}N_{10}O_{12}S$
[Leu^1, NMeSer^7]MC-LR	1055.6136	$C_{52}H_{83}N_{10}O_{13}$
**Unknown 3**		
[D-Asp^3, ADMAdda^5, Dha^7]MC-HilR<br>[Gly^1, Asp^3, ADMAdda^5, Dhb^7]MC-LHAr<br>[d-Asp^3, ADMAdda^5]MC-LR<br>[ADMAdda^5, Dha^7]MC-LR<br>[Asp^3, ADMAdda^5, Dhb^7]MC-LR	1009.5353	$C_{49}H_{73}N_{10}O_{13}$
[MeAla^1]MC-LR or [MeLeu^2]MC-LR<br>MC-HilR<br>MC-Lhar<br>[d-Glu(OCH_3)$_6$]MC-LR<br>[Mdhb^7]MC-LR<br>[Leu^1, Asp^3, DMAdda^5]MC-LR	1009.5717	$C_{50}H_{77}N_{10}O_{12}$

Table S2. Major fragments of MC-LR detected in the standard solution at a RT of 2.84 min. *m/z* is presented as detected under positive ionization mode without mass correction.

Fragment Structure	Molecular Formula	*m/z* (Da)
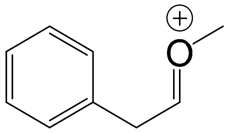	$C_9H_{11}O$ *(ADDA moiety)*	135.0808
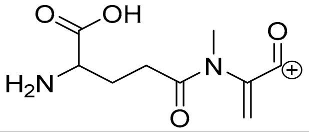	$C_9H_{13}N_2O_4$	213.0799
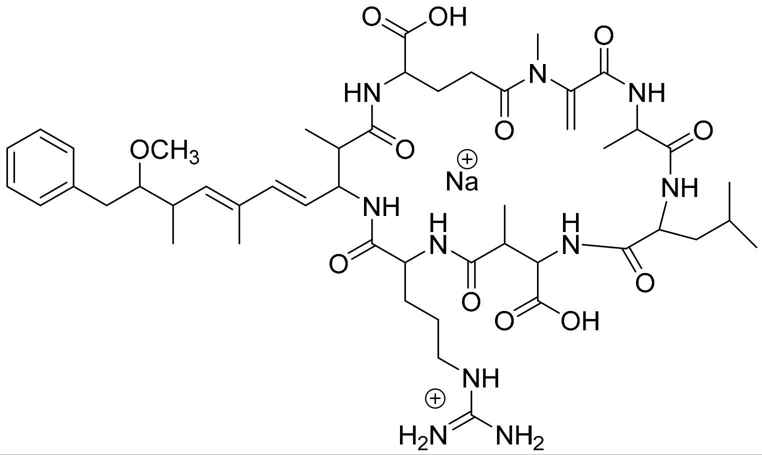	$C_{49}H_{75}N_{10}NaO_{12}$	509.2528
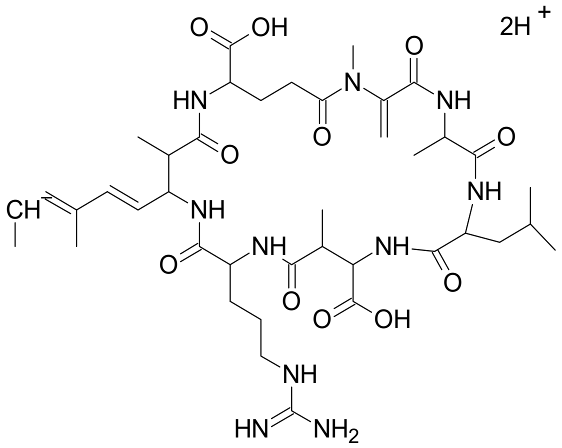	$C_{40}H_{63}N_{10}O_{11}$	861.4587

Table 3. Major fragments of cyanopeptolin CPT911 detected in the samples at RT 2.13 min and 2.25 min. *m*/*z* is presented as detected under positive ionization mode without mass correction.

Fragment Structure	Molecular Formula	RT (min)	*m*/*z*
	$C_5H_{12}N$	2.25	86.0940
	$C_6H_{13}N_4O$	2.13	157.0983
		2.25	157.0983

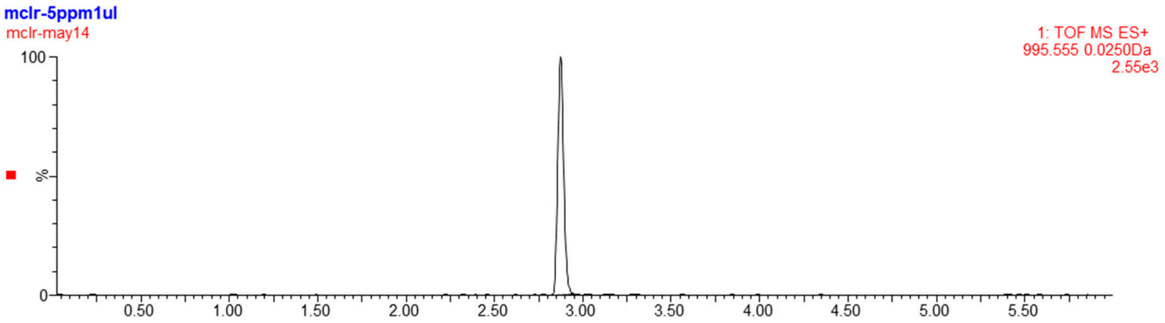

(**a**)

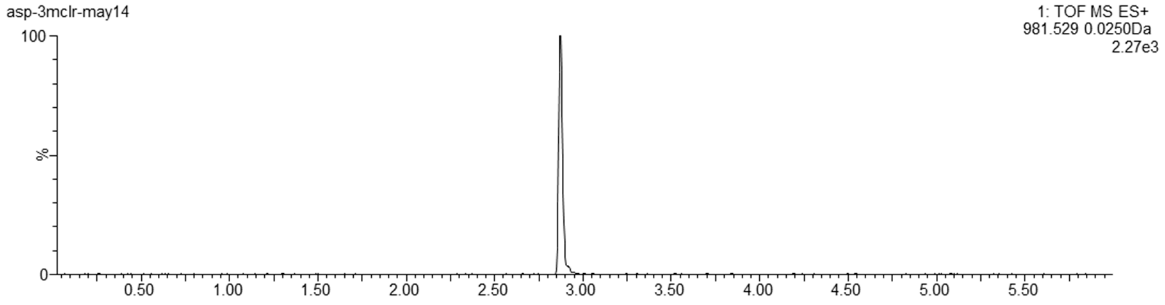

(**b**)

Figure S1. Separation of standards in a positive ionization mode (**a**) MC-LR (2.87 min); (**b**) [Asp^3]-MC-LR (2.88 min). In this case, fragmentation is the only way to differentiate between the two metabolites.

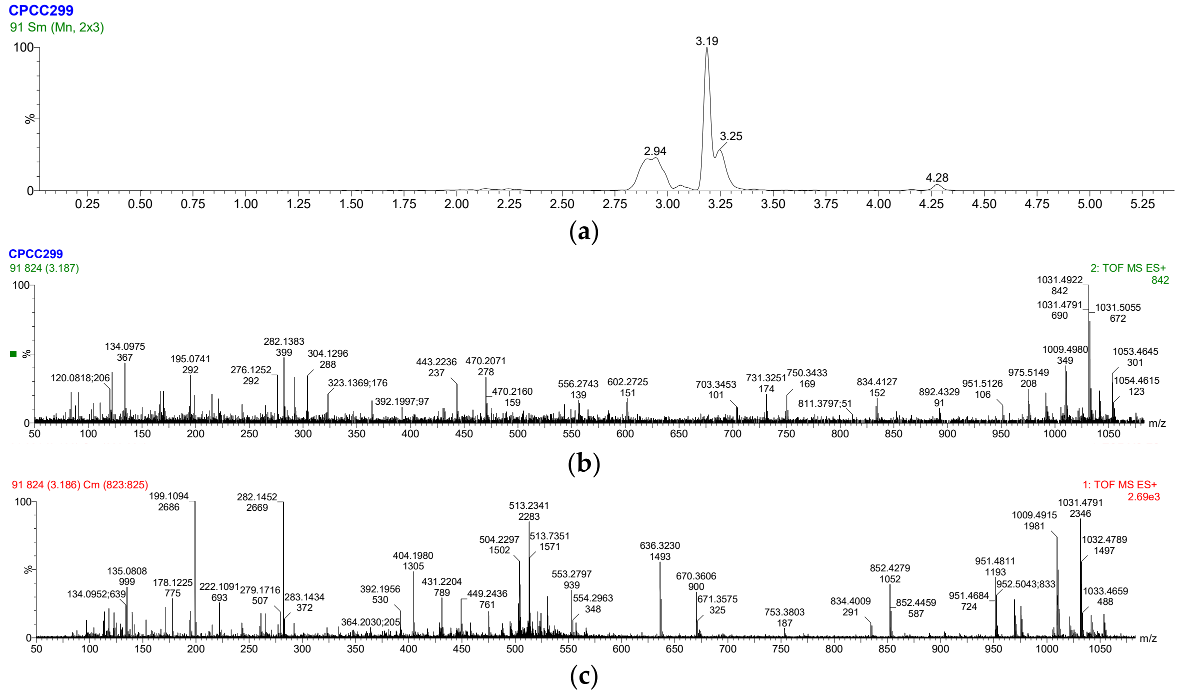

Figure S2. Unknown 1 separation chromatogram at RT 3.19 min (**a**), and spectra under high energy (**b**) and low energy (**c**) collision in positive ionization mode. The m/z of 134.0975 Da corresponds to the mass of the ADDA moiety present in all microcystins.

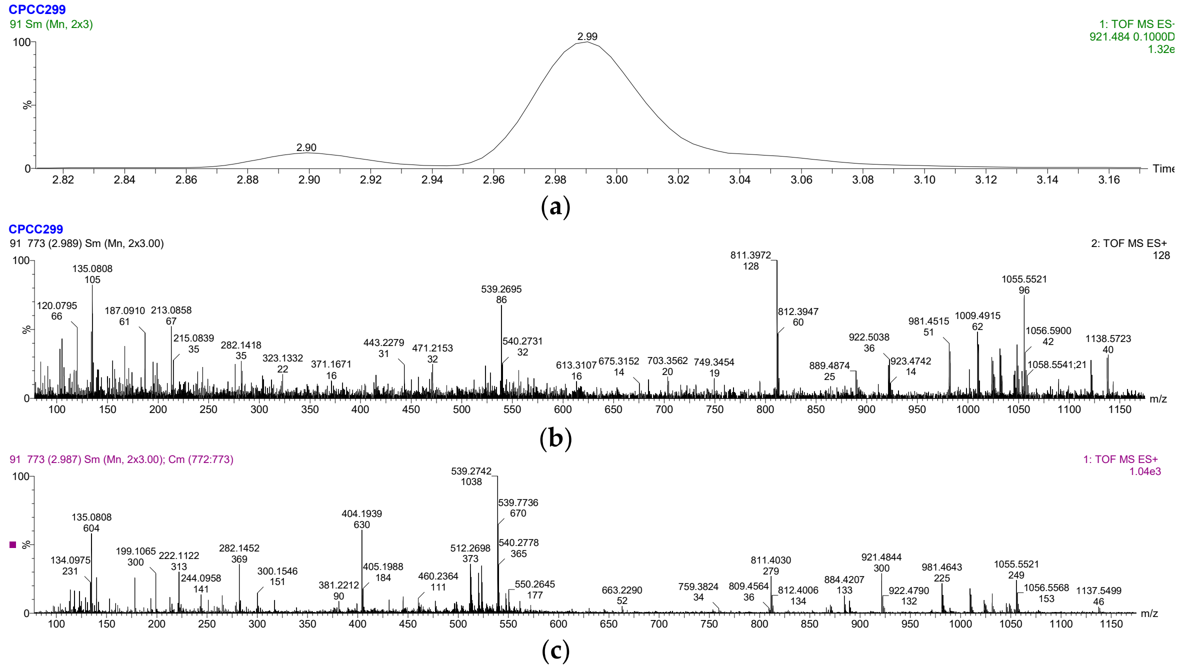

Figure S3. Unknown 2 separation chromatogram at RT 2.98 min (**a**), and spectra under high energy (**b**) and low energy (**c**) collision in positive ionization mode. The m/z of 135.0808 Da corresponds to the fragment mass of the ADDA moiety present in all microcystins.

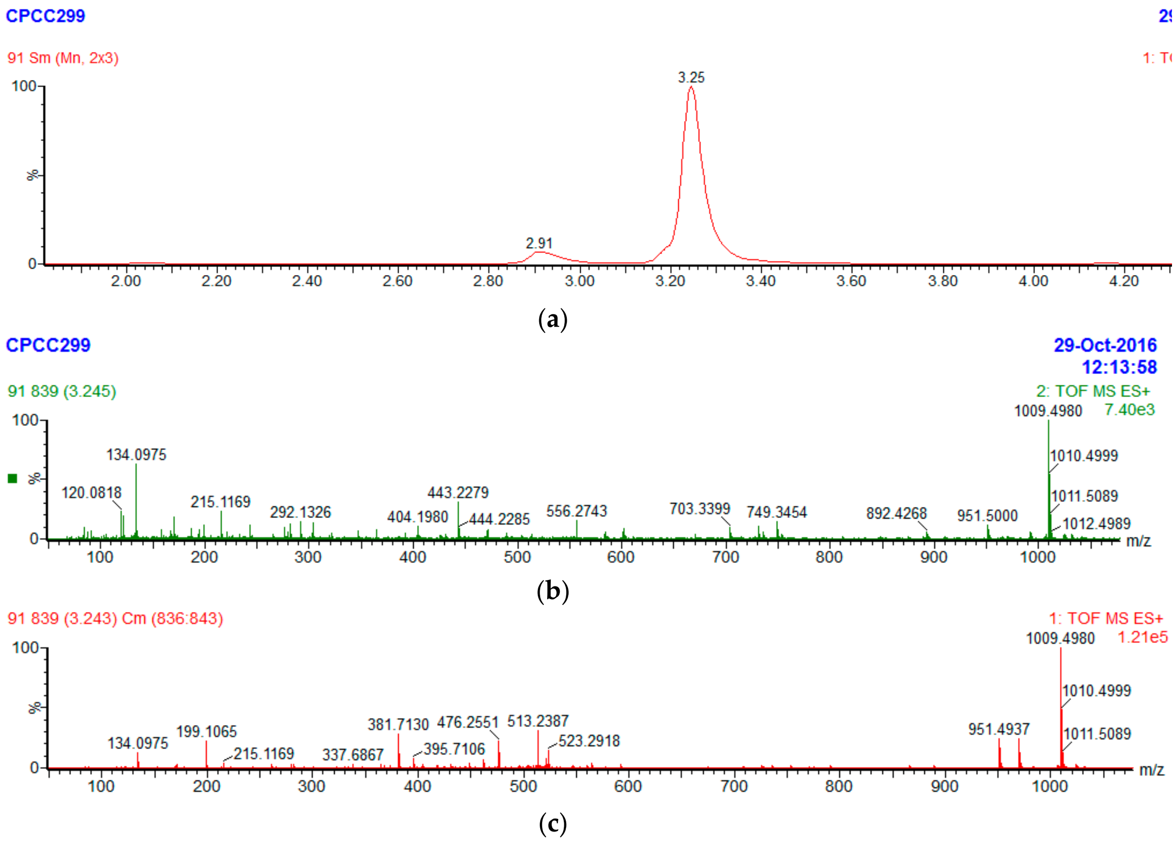

Figure S4. Unknown 3 separation chromatogram at RT 3.34 min (**a**), and spectra under high energy (**b**) and low energy (**c**) collision in positive ionization mode. The m/z of 135.0975 Da corresponds to the fragment mass of the ADDA moiety present in all microcystins.

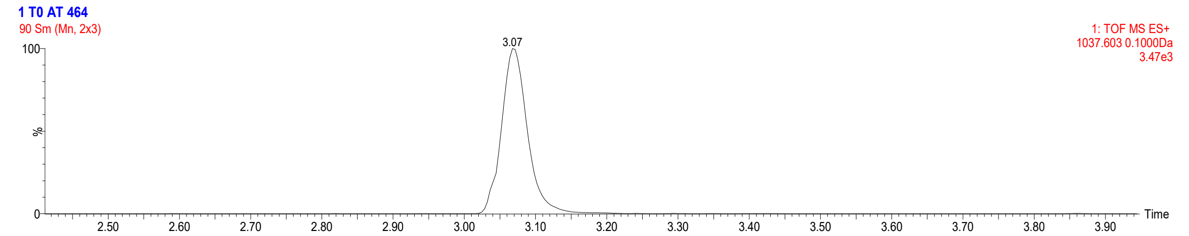

(**a**)

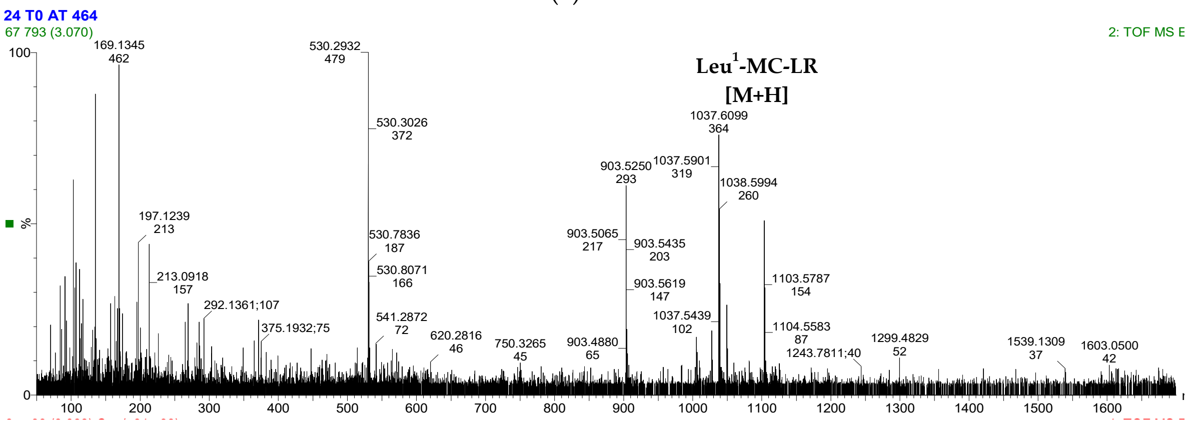

(**b**)

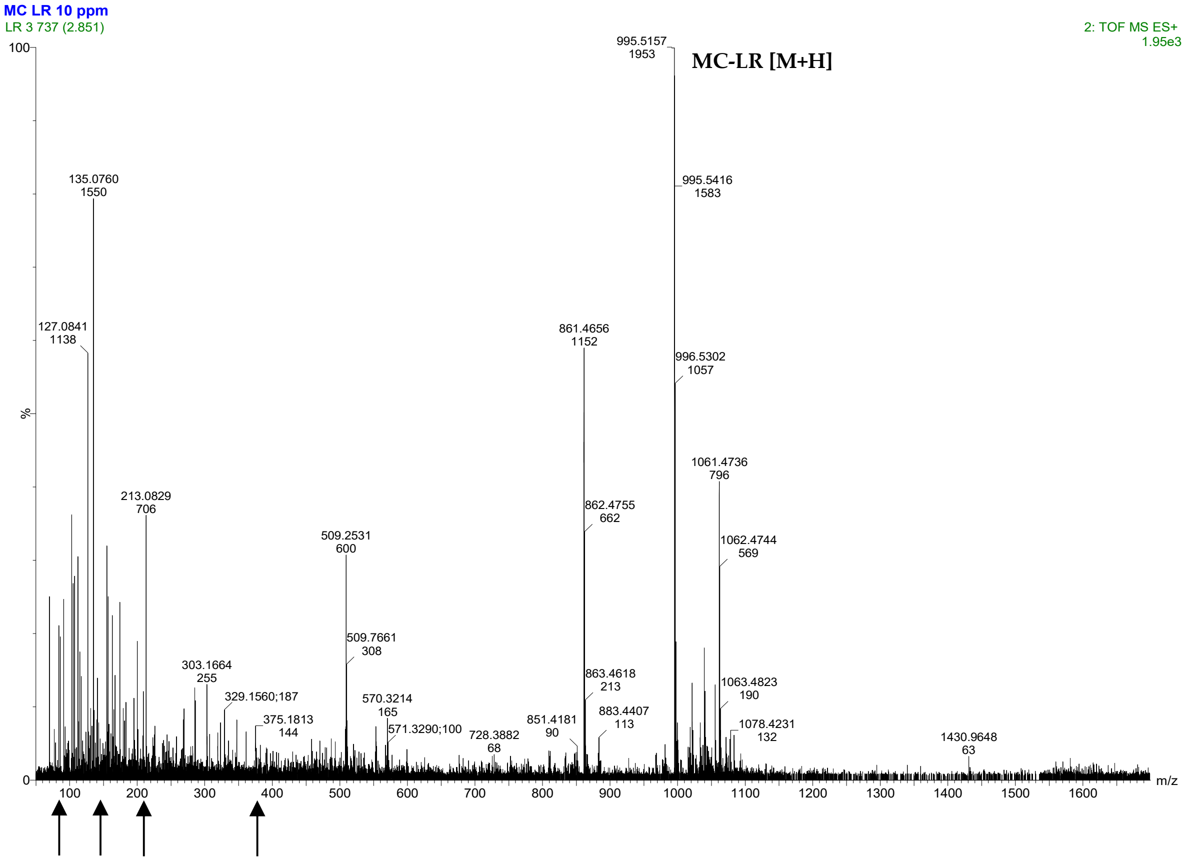

(**c**)

Figure S5. (**a**) Chromatography of [Leu¹]-MC-LR variant; (**b**) high energy spectrum of [Leu¹]-MC-LR; (**c**) high-energy spectrum of the standard solution of MC-LR. The arrows point to the common fragments between the two molecules.

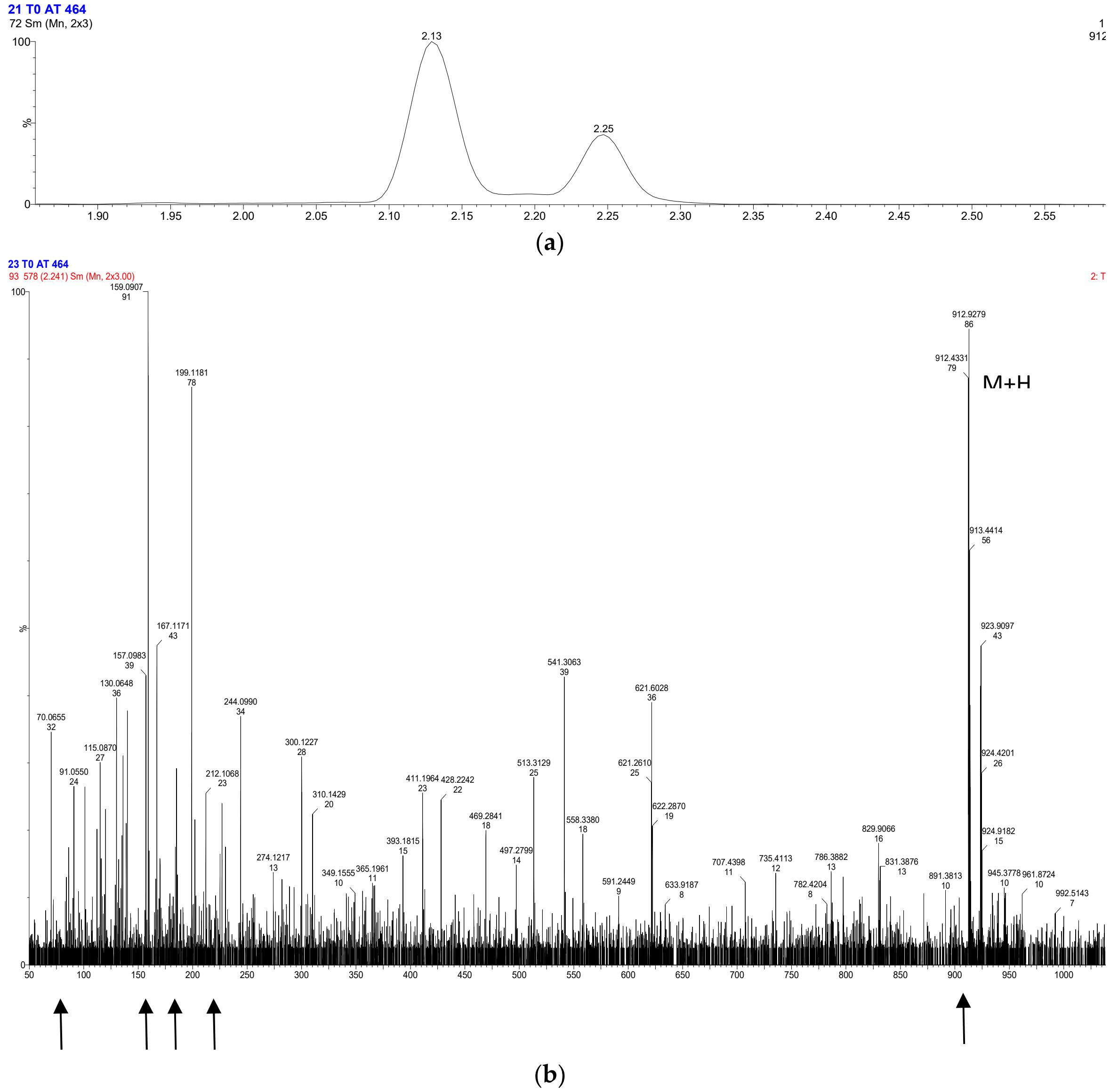

Figure S6. CPT911 detection in *Microcystis aeruginosa* strain CPCC464; (**a**) Separation of two isomers, CPT911A at RT 2.13 min and CPT911B at RT 2.25 min; (**b**) High energy spectra for CPT911B. Signals corresponding to identified fragments are indicated by an arrow. The pseudo molecule [M + H] represents the signal of the mass of the whole molecule under positive ionization mode.
